# A bibliometric analysis of synaptic plasticity and epilepsy from 2003 to 2023

**DOI:** 10.3389/fneur.2025.1533268

**Published:** 2025-07-16

**Authors:** Xuewei Li, Huaiyu Sun, Zhiqing Chen, Hongmei Meng, Daguang Zhang

**Affiliations:** ^1^Department of Radiology, First Hospital of Jilin University, Jilin, China; ^2^Department of Neurology, First Hospital of Jilin University, Jilin, China; ^3^Department of Bone and Joint Surgery, Orthopaedics Clinic, The First Hospital of Jilin University, Jilin, China

**Keywords:** epilepsy, synaptic plasticity, bibliometric analysis, visualization, VOSviewer, CiteSpace

## Abstract

**Background and purpose:**

Epilepsy is a prevalent and chronic neurological disorder. Recent studies suggest that synaptic plasticity could be a promising therapeutic target for epilepsy. This research employed bibliometric techniques to assess the study trends of synaptic plasticity in epilepsy over the last 20 years, highlighting major areas of focus and new topics.

**Methods:**

Research articles on synaptic plasticity in epilepsy, spanning 2003 to 2023, were sourced from the Web of Science Core Collection (WoSCC) database. Tools including CiteSpace, VOSviewer, and various online bibliometric platforms were utilized to conduct a deeper analysis of the collected data.

**Results:**

From 2003 to 2023, a total of 1,060 publications related to synaptic plasticity in epilepsy were indexed, including 309 review articles. Over the past two decades, both the number of publications and their citations have increased. The United States emerged as the leading country in terms of the number of both review and original research articles published, highlighting its significant influence in this field. Among all authors, Fabio Benfenati was the most cited in review articles, while Xuefeng Wang was the most cited in original research articles. Over the past 20 years, Frontiers in Cellular Neuroscience published the highest number of review articles on synaptic plasticity in epilepsy, while The Journal of Neuroscience published the most original research articles on this topic.

**Conclusion:**

This research examined 1,369 studies on synaptic plasticity in epilepsy and highlighted the prevailing trends in the field. The research findings indicate that the current focus of review studies is on gamma-aminobutyric acid, amyloid beta peptide, and glutamate receptors, while the focus of original research is on astrocytes, NMDA receptors, and long-term potentiation.

## 1 Introduction

Epilepsy is a devastating and complex neurological disease that impacts approximately 70 million individuals globally (1, 2). At present, medications for epilepsy administered in medical practice can manage seizures and reduce seizure burden, but they only lead to seizure freedom in about one third of cases. Moreover, these medications do not alter the underlying disease mechanism or the long-term prognosis of epilepsy. Currently, the only curative procedure for epilepsy is surgery to remove the epileptogenic zone (3). Consequently, it is crucial to identify novel treatment targets promptly.

Epilepsy frequently causes cognitive deficits, particularly in learning and memory, with synaptic plasticity serving as the foundational structure for these functions (4). Temporal lobe epilepsy (TLE) is the predominant type of focal epilepsy in humans, impacting several brain regions responsible for memory formation and retention, especially the hippocampus (5, 6). Cognitive impairments, such as memory loss, are observed in numerous animal models of TLE (7, 8). The efficiency of synapses can either increase or decrease for an extended period, influenced by the specific pattern of synaptic input activation (9). These processes are known as long-term potentiation (LTP) and long-term depression (LTD) (10, 11). LTP and LTD are often suggested as fundamental cellular mechanisms for learning and memory (12, 13). LTP aids in memory creation, while LTD can deactivate memories (14). As a result, LTP and LTD work in tandem to alter synaptic strength, thereby encoding memories. Therefore, as the effectiveness of LTP diminishes, the patients’ cognitive abilities tend to decline.

Bibliometric analysis serves as a crucial statistical technique for quantitatively examining extensive and diverse sets of publications (15). This technique can quantitatively evaluate the shape distribution, connections, and grouping within a research area, and it has emerged as a widely used method for determining the credibility, quality, and influence of scholarly work (16).

In the last 20 years, numerous studies have demonstrated the link between synaptic plasticity and epilepsy. However, to the best of our knowledge, there has not been a bibliometric analysis on this topic. To address this gap, this study utilized the Web of Science Core Collection (WoSCC). We retrieved bibliometric data (annual articles, countries/regions, authors, institutions, disciplines, journals, references, and keywords) for each synaptic plasticity and epilepsy research field and performed descriptive statistics. This article provides a comprehensive overview of the research landscape on synaptic plasticity and epilepsy from 2003 to 2023, utilizing CiteSpace and VOSviewer to create knowledge maps that systematically analyze publication trends, citation patterns, and authorship networks. This study aims to identify key research areas, emerging trends, and influential contributions in the field.

## 2 Methods

### 2.1 Data source and literature search strategy

**Figure 1:**
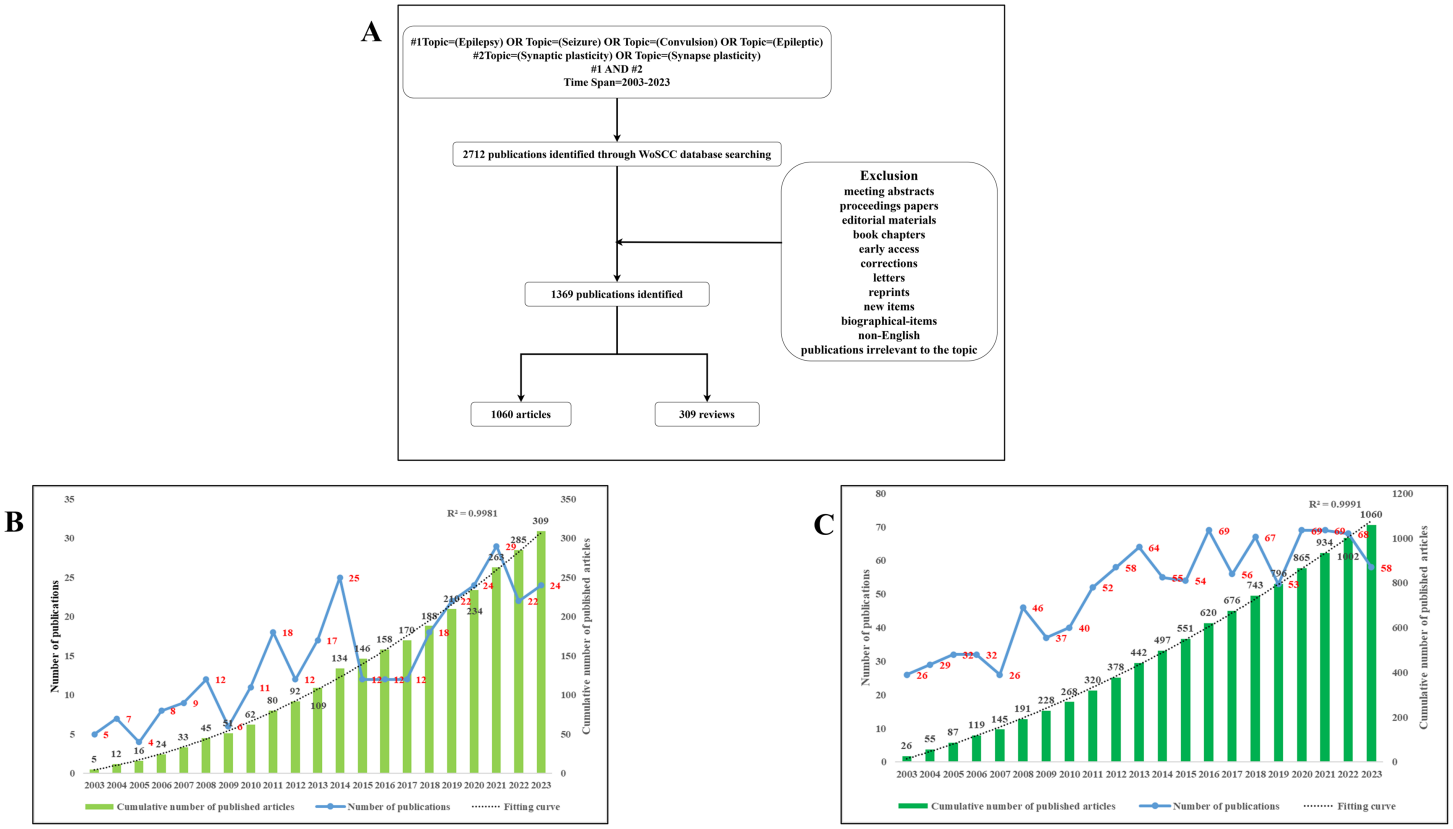
**(A)** Diagram outlining the research process. This figure was drawn by Figdraw. **(B)** Annual publication count and the total accumulated number of review publications related to synaptic plasticity in epilepsy. **(C)** Annual publication count and the total accumulated number of original research publications related to synaptic plasticity in epilepsy.

We conducted a comprehensive search of research published between January 1, 2003, and December 31, 2023, covering relevant literature within this period from the Science Citation Index Expanded (SCI-Expanded) in the Web of Science Core Collection (WoSCC). The search criteria were as follows: TS = (“Epilepsy” OR “Seizure” OR “Convulsion” OR “Epileptic”) AND TS = (“synaptic plasticity” OR “synapse plasticity”). To ensure a thorough analysis, only peer-reviewed English-language articles were considered. After a detailed review of all the documents, a total of 1,369 valid references were identified (see Figure 1A).

### 2.2 Bibliometric analysis

Bibliometric data for the review and original research were analyzed separately using VOSviewer (version 1.6.20) and CiteSpace (version 6.1. R3).

## 3 Results

### 3.1 Overview of publication status

A total of 1,369 publications, comprising 1,060 articles and 309 reviews, met the inclusion criteria. The annual distribution of review and original research publications on synaptic plasticity in epilepsy is illustrated in Figures 1B,C. The line chart depicts a gradual increase in publications on synaptic plasticity in epilepsy over the 20-year period from 2003 to 2023. The growth rate of publications from 2013 to 2023 was slower compared to the period from 2003 to 2013.

### 3.2 Bibliometric analysis of countries

To better understand the international distribution of academic output, the dataset was reanalyzed by categorizing the publications into two types: reviews and original research articles. Publication counts were then assessed separately for each category. The leading ten nations with the highest publication output were evaluated according to the total number of works produced by all contributors. The number of publications was indicated by the size of the circles, so countries with a higher volume of articles typically had larger circles. Connections between the two nations have led to the joint publication of articles.

**Figure 2:**
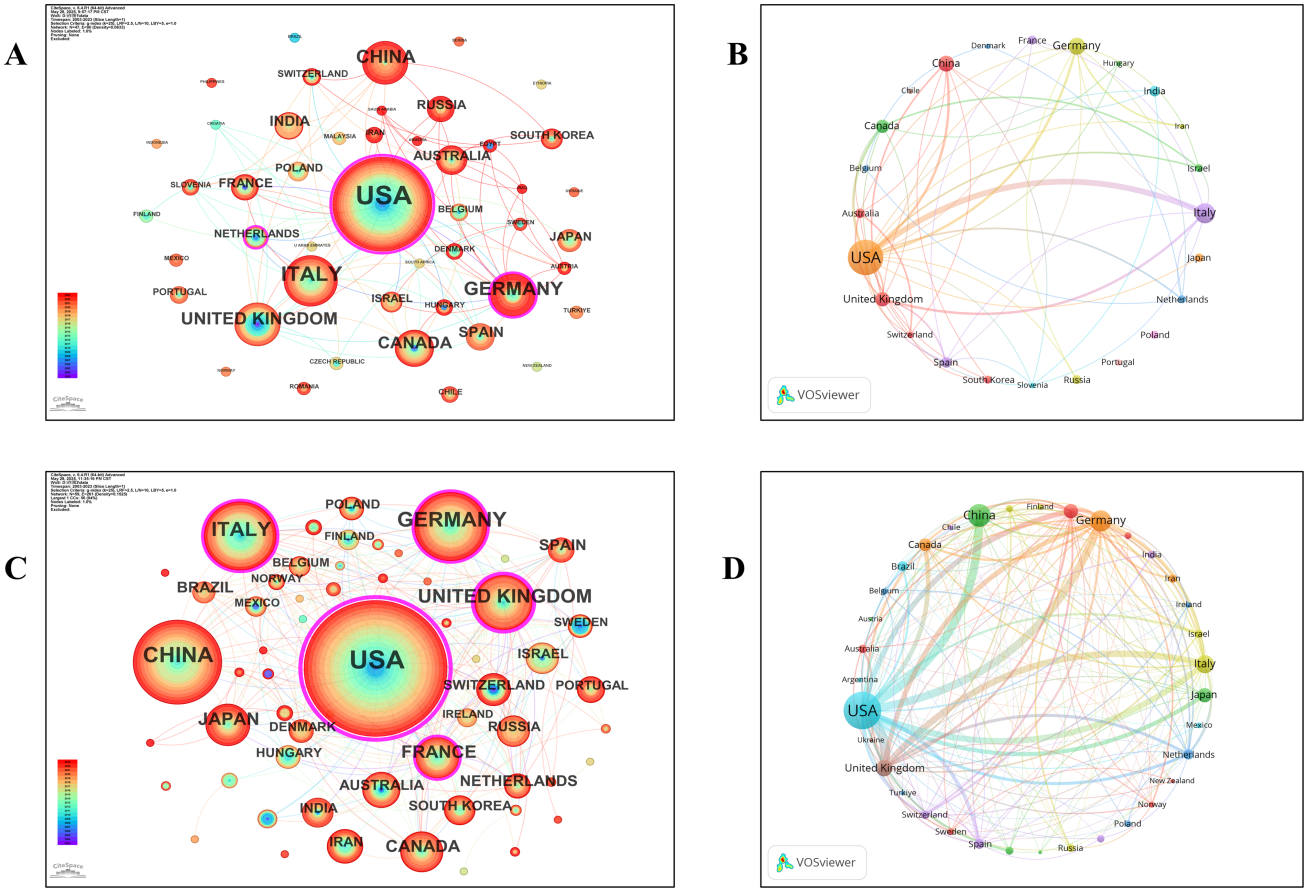
Cooperation map of countries/regions on synaptic plasticity in epilepsy. **(A,B)** A visual map for the CiteSpace network and the VOSviewer network of review publications. **(C,D)** A visual map for the CiteSpace network and the VOSviewer network of original research publications.

The United States published the highest number of both review and original research articles, with 120 and 405 publications, respectively. Italy and the United Kingdom ranked second and third in terms of review article output, contributing 39 and 20 publications, respectively. China was the second-largest contributor of original research articles, with 151 publications, followed by Germany with 129 (Tables 1, 2; Figure 2). In terms of citations, the United States also led in both categories, receiving 9,411 citations for review articles and 21,221 citations for original research articles. Italy (2,830 citations) and the United Kingdom (2,195 citations) ranked second and third for review article citations. For original research articles, Germany (4,359 citations) and China (3,476 citations) held the second and third positions, respectively. Notable collaborative partnerships were established among the United States, Italy, the United Kingdom, China, and Germany (Figures 2B,D).

**Table 1:** Leading ten nations with the highest volume of review publications on synaptic plasticity in epilepsy.

Rank	Countries	Counts	Total link strength	Citations
1	USA	120	32	9,411
2	Italy	39	16	2,830
3	Germany	28	13	1,468
4	China	25	10	1,076
5	United Kingdom	20	15	2,195
6	Canada	17	9	1,245
7	Spain	11	8	799
8	India	11	3	832
9	Australia	9	8	466
10	France	8	4	866

**Table 2:** Leading ten nations with the highest volume of original research publications on synaptic plasticity in epilepsy.

Rank	Countries	Counts	Total link strength	Citations
1	USA	405	208	21,221
2	China	151	47	3,476
3	Germany	129	121	4,359
4	Italy	98	81	3,421
5	United Kingdom	78	118	3,260
6	France	59	78	2,408
7	Japan	59	27	1704
8	Canada	48	50	1,419
9	Netherlands	32	49	1,173
10	Spain	31	36	1,090

### 3.3 Bibliometric analysis of institutions

Johns Hopkins University ranked first in the number of review articles published, with a total of 8 papers, followed by the Russian Academy of Sciences and the University of Genoa, each with 6 review articles (Table 3). Chongqing Medical University, the University of Pennsylvania, and Baylor College of Medicine were the top three institutions in terms of original research article output, with 31, 20, and 18 publications, respectively (Table 4).

**Table 3:** Leading ten institutions with the highest volume of review publications on synaptic plasticity in epilepsy.

Rank	Institutions	Publications
1	Johns Hopkins University	8
2	Russian Academy of Sciences	6
3	University of Genoa	6
4	Yale University	6
5	University of Pennsylvania	5
6	University of Toronto	5
7	Children’s Hospital of Philadelphia	4
8	Polish Academy of Sciences	4
9	Ruhr University Bochumn	4
10	University College London	4

**Table 4:** Leading ten institutions with the highest volume of original research publications on synaptic plasticity in epilepsy.

Rank	Institutions	Publications
1	Chongqing Medical University	31
2	University of Pennsylvania	20
3	Baylor College of Medicine	18
4	University of Genoa	18
5	University College London	15
6	University of Münster	15
7	INSERM – Institut National de la Santé et de la Recherche Médicale	14
8	Istituto Italiano di Tecnologia	14
9	Università Vita-Salute San Raffaele	14
10	Duke University	13

**Figure 3:**
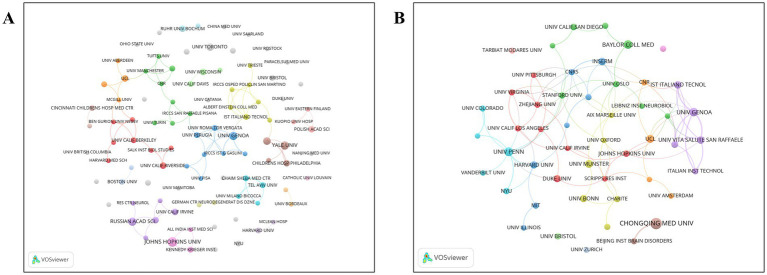
**(A)** Collaboration networks among institutions of review publications on synaptic plasticity in epilepsy. **(B)** Collaboration networks among institutions of original research publications on synaptic plasticity in epilepsy.

Figure 3A presents the institutional collaboration network for review articles, generated using VOSviewer, while Figure 3B illustrates the institutional collaboration network for original research articles.

### 3.4 Bibliometric analysis of authors

Tables 5, 6 respectively provide detailed abstracts of the top ten authors of the review and the original study, including their names, publication counts, and the average citation count per paper.

**Table 5:** The leading researchers of review publications on synaptic plasticity in epilepsy.

Rank	Author	Publications	Citations	Average citation
1	Reddy, Doodipala Samba	5	440	88.00
2	Benfenati, Fabio	4	194	48.50
3	Binder, Devin K.	3	209	69.67
4	Johnston, Michael V.	3	389	129.67
5	Stafstrom, Carl E.	3	174	58.00
6	Costa, Cinzia	3	100	33.33
7	Curatolo, Paolo	3	192	64.00
8	Maggio, Nicola	3	109	36.33
9	Corradi, Anna	2	66	33.00
10	Fassio, Anna	2	103	51.50

**Table 6:** The leading researchers in the area of original research publications on synaptic plasticity in epilepsy.

Rank	Author	Publications	Citations	Average citation
1	Wang, Xuefeng	19	264	13.89
2	Benfenati, Fabio	15	772	51.47
3	Valtorta, Flavia	13	694	53.38
4	Baldelli, Pietro	9	519	57.67
5	Jensen, Frances E.	9	413	45.89
6	Wang, Liang	8	262	32.75
7	Xiao, Fei	8	136	17.00
8	Zhang, Yanke	7	78	11.14
9	Luo, Jing	6	172	28.67
10	Ma, Yuanlin	6	85	14.17

**Figure 4:**
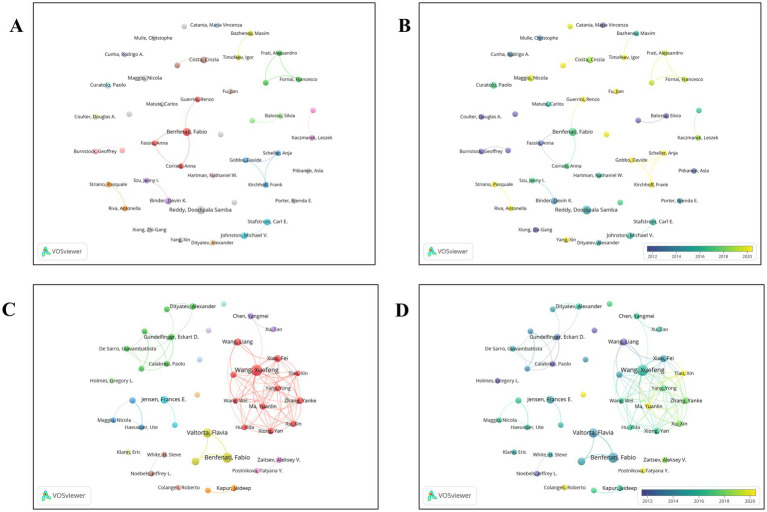
Distribution of authors. **(A)** A map showing the connections between the authors (review publications). **(B)** Dynamics and trends of authors over time (review publications). **(C)** A map showing the connections between the authors (original research publications). **(D)** Dynamics and trends of authors over time (original research publications).

A co-authorship network was visualized for the top 45 authors of review articles by including those with at least two published reviews and merging author name variants using VOSviewer. Among these 45 authors, 19 had no connections with others in the network. The largest group, shown in red, consisted of 4 authors (Figure 4A). Most of these review articles were published after 2018 (Figure 4B).

Similarly, a co-authorship network was constructed for the top 42 authors of original research articles, including those with at least five published articles and merging synonyms in VOSviewer. Among these 42 authors, 7 had no connections with other authors. The largest group, represented in red, comprised 12 authors and formed the most substantial cluster, demonstrating strong collaborative potential with a total of 86 publications (Figure 4C). Most of these articles were also published after 2018 (Figure 4D).

### 3.5 Bibliometric analysis of journals

Bibliometric analysis of reviews identified 159 distinct journals publishing review articles related to synaptic plasticity in epilepsy. Table 7 summarizes the top ten journals contributing to this topic. Frontiers in Cellular Neuroscience had the highest output (*n* = 19, 6.1%), followed by International Journal of Molecular Sciences (*n* = 15, 4.9%), Frontiers in Molecular Neuroscience (*n* = 14, 4.5%), Epilepsy & Behavior (*n* = 7, 2.3%), and Molecular Neurobiology (*n* = 7, 2.3%). Supplementary Figure 1A illustrates numerous cross-disciplinary links among journals through dual-map overlays (journals citing articles are on the left, while cited journals are on the right; the connecting lines represent citation relationships). A primary citation pathway was identified: articles published in molecular/biological/immunological journals were predominantly cited by those in molecular/biological/genetic journals.

**Table 7:** Lists the leading 10 primary journals of review publications on synaptic plasticity in epilepsy.

Rank	Journal	Documents
1	Frontiers in Cellular Neuroscience	19
2	International Journal of Molecular Sciences	15
3	Frontiers in Molecular Neuroscience	14
4	Epilepsy Behavior	7
5	Molecular Neurobiology	7
6	Frontiers in Neurology	6
7	Neural Plasticity	6
8	Neuropharmacology	6
9	Neuroscience	6
10	Progress in Neurobiology	5

Bibliometric analysis of original research identified 200 distinct journals publishing original research articles related to synaptic plasticity in epilepsy. Table 8 summarizes the top ten journals contributing to this topic. The Journal of Neuroscience had the highest output (*n* = 72, 6.7%), followed by Neuroscience (*n* = 43, 4.1%), Epilepsia (*n* = 37, 3.5%), Neurobiology of Disease (*n* = 34, 3.2%), and Brain Research (*n* = 29, 2.7%). Supplementary Figure 1B demonstrates numerous cross-disciplinary links among journals using dual-map overlays (citing journals are on the left, while cited journals are on the right; the connecting lines illustrate citation relationships). A primary citation pathway was identified: articles published in molecular/biological/immunological journals were predominantly cited by those in molecular/biological/genetic journals.

**Table 8:** Lists the leading 10 primary journals of original research publications on synaptic plasticity in epilepsy.

Rank	Journal	Documents
1	Journal of Neuroscience	72
2	Neuroscience	43
3	Epilepsia	37
4	Neurobiology of Disease	34
5	Brain Research	29
6	European Journal of Neuroscience	24
7	PLoS One	22
8	Cerebral Cortex	19
9	Epilepsy Research	18
10	Hippocampus	18

### 3.6 Co-citation analysis on cited reference

A co-citation map of review articles was generated using VOSviewer. In the subsequent citation analysis, a total of 60 references were identified. Supplementary Table 1 lists the top ten most frequently cited review references related to synaptic plasticity in epilepsy research. The number of co-citations ranged from 8 to 19. Supplementary Figure 2A illustrates the co-citation network of frequently cited papers, which is divided into four main clusters, each marked with a distinct color.

Subsequently, a co-citation map of original research articles was created. In this citation analysis, a total of 86 references were identified. Supplementary Table 2 presents the top ten most frequently cited original research references related to synaptic plasticity in epilepsy research. The number of co-citations ranged from 15 to 148. Supplementary Figure 2B shows the co-citation network of frequently cited original research papers, which is also divided into four major clusters, each represented by a unique color.

### 3.7 Co-citation analysis on cited journals

For the review articles, we selected 78 journals for a co-citation analysis. The results yielded a network map comprising three distinct clusters (Supplementary Figure 2C). The top three most frequently cited journals were The Journal of Neuroscience (cited 3,518 times), Neuron (cited 1,744 times), and Proceedings of the National Academy of Sciences of the United States of America (cited 1,379 times) (Supplementary Table 3).

From among the original research articles we selected 98 journals for a co-citation analysis. This study generated a network map comprising four distinct clusters (Supplementary Figure 2D). The top three most frequently cited journals were The Journal of Neuroscience (cited 5,898 times), Neuron (cited 3,014 times), and Proceedings of the National Academy of Sciences of the United States of America (cited 2,016 times) (Supplementary Table 4).

### 3.8 An analysis of keywords

**Figure 5:**
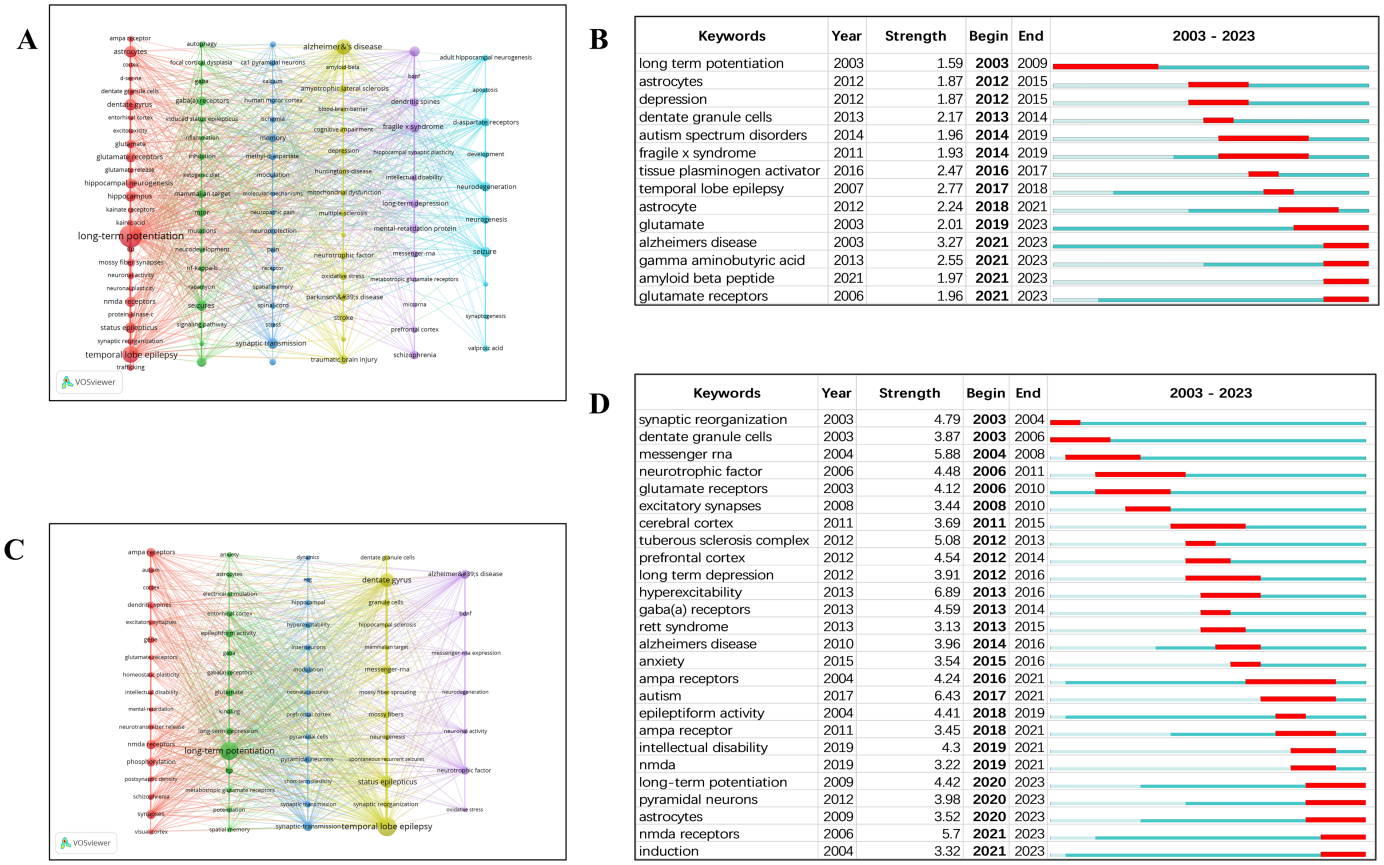
**(A)** Keyword network mapping (review publications). **(B)** Top 14 keywords with strongest citation bursts (review publications). **(C)** Keyword network mapping (original research publications). **(D)** Top 26 keywords with strongest citation bursts (original research publications).

A total of 101 keywords were extracted from the 309 review articles. Only 34.7% of these keywords appeared more than 10 times, while a large proportion (approximately 25.7%) appeared only five times. This stark contrast indicates that only a small subset of keywords were used frequently. Based on the network visualization of the 101 most frequently used keywords, six distinct clusters were identified (Figure 5A).

All keywords that experienced citation bursts first appeared in 2003 (Figure 5B). Recently, terms such as “glutamate,” “Alzheimer’s disease,” “amyloid beta peptide,” and “gamma aminobutyric acid” have emerged as research frontiers. Table 9 provides a detailed overview of the occurrence rates of the 20 most common keywords. Notably, terms such as “long-term potentiation,” “temporal-lobe epilepsy,” and “Alzheimer’s disease” have remained consistently significant.

**Table 9:** The 20 most frequent keywords of review publications on synaptic plasticity in epilepsy.

Rank	Keywords	Occurrences
1	Long-term potentiation	85
2	Temporal lobe epilepsy	46
3	Alzheimer’s disease	38
4	Astrocytes	22
5	Dentate gyrus	22
6	Fragile x syndrome	21
7	Hippocampus	20
8	Seizures	20
9	Status epilepticus	20
10	Synaptic transmission	20
11	Autism spectrum disorders	18
12	Hippocampal neurogenesis	18
13	NMDA receptors	18
14	Seizure	18
15	Glutamate receptors	17
16	Mental-retardation protein	16
17	Neurodegeneration	16
18	Traumatic brain injury	16
19	Tuberous sclerosis complex	15
20	Dendritic spines	14

From the 1,060 original research articles, 65 keywords were extracted. Only 13.9% of these keywords appeared more than 14 times, whereas a large portion (approximately 38.7%) appeared only twice. This again illustrates that only a limited number of keywords were frequently used. Based on the network visualization of the 65 most common keywords, five distinct clusters were identified (Figure 5C).

All keywords with citation bursts also initially appeared in 2003 (Figure 5D). Recently, keywords such as “long-term potentiation,” “pyramidal neurons,” “astrocytes,” and “NMDA receptors” have emerged as leading research topics. Table 10 presents a detailed summary of the occurrence frequencies of the 20 most common keywords. It is noteworthy that terms like “temporal-lobe epilepsy,” “long-term potentiation,” and “dentate gyrus” have consistently held significant importance.

**Table 10:** The 20 most frequent keywords in studies of original research publications on synaptic plasticity in epilepsy.

Rank	Keywords	Occurrences
1	Temporal lobe epilepsy	217
2	Long-term potentiation	208
3	Dentate gyrus	119
4	Status epilepticus	90
5	NMDA receptors	58
6	Synapses	56
7	Synaptic-transmission	53
8	Messenger-RNA	51
9	Alzheimer’s disease	50
10	AMPA receptors	50
11	Phosphorylation	48
12	Neurotrophic factor	41
13	Epileptiform activity	36
14	Glutamate	36
15	Mossy fibers	35
16	Granule cells	34
17	LTP	34
18	Long-term depression	33
19	Dendritic spines	32
20	Gene	32

## 4 Discussion

This research gathered studies from the WoSCC database that concentrate on studying synaptic plasticity in the context of epilepsy. A total of 1,369 references from 587 journals were included and analyzed. In American journals, numerous research papers have been published and cited simultaneously. Over the last 20 years, bibliometric analysis of studies on synaptic plasticity in epilepsy revealed a steady rise in publications, suggesting growing interest in this area.

A bibliometric study on synaptic plasticity’s involvement in epilepsy research over the last 20 years revealed a steady rise in the number of articles published, suggesting growing interest in this area. The findings indicate that the primary contributors in this area are the United States, China, Italy, and Germany (Figure 2). Our examination of the publication counts and citation metrics across various institutions revealed that the University of California was the most prolific. Additionally, we identified that the United States and Italy collaborated most often due to the strong academic interactions between their researchers. Nonetheless, the quantity of published research and citations in China remains inadequate, and international collaboration is limited, suggesting that Chinese scientists should enhance partnerships with other nations to boost their publication and citation rates.

We evaluated the impact of authors in the domain by ranking them according to their publication count and overall citations. Data from WoSCC revealed that Benfenati Fabio secured the top position with the highest citation count, followed by Valtorta Flavia and Reddy Doodipala Samba. According to the number of articles published, Wang Xuefeng ranked first with 19 articles.

Examining well-known publications can offer scientists a clear path for investigation in this field of study. Our research revealed that the Journal of Neuroscience has the highest number of publications and leads in citation rankings. Researchers can easily identify appropriate journals for their papers by referring to the journals’ rankings.

By examining the chronological distribution of relevant references and the sudden increase in keyword citations, we can identify key areas, emerging trends, and developments within this scientific research field. A comparison of burst keywords from review articles and original research revealed a notable pattern: certain keywords, such as “long-term potentiation” and “astrocytes,” showed citation bursts in review articles as early as 2003 or earlier, whereas in original research, these same keywords did not exhibit bursts until 2020.

This discrepancy may be attributed to the nature of review articles, which are fundamentally designed to summarize, synthesize, and anticipate research trends based on existing studies. For example, although empirical research on LTP was still limited in the early stages, its potential significance as a core mechanism in synaptic plasticity may have already been highlighted and disseminated by expert authors through review publications. Moreover, high-impact reviews are often authored by leading experts or well-established research teams in the field, who typically possess a forward-looking academic vision. These authors are capable of promoting novel concepts through reviews even before such ideas are widely accepted or substantiated by experimental data. In addition, early-stage empirical studies on LTP may have been constrained by technical limitations—such as challenges in electrophysiology or optogenetics—which made direct investigation difficult. Only with the advancement of experimental tools (e.g., refined methods for precisely monitoring synaptic activity in the hippocampus) in recent years did a substantial volume of research emerge, resulting in the delayed burst of these keywords in original research literature.

An intriguing finding of this bibliometric analysis is the emergence of “Alzheimer’s disease” as a burst keyword in review articles related to synaptic plasticity and epilepsy. Although Alzheimer’s disease (AD) is not traditionally categorized within the domain of epilepsy research, its appearance as a frequently cited term highlights a growing interdisciplinary convergence between neurodegenerative and epileptic disorders. One plausible explanation lies in the shared pathophysiological mechanisms between the two conditions. Both epilepsy and AD are closely linked to disruptions in synaptic plasticity, particularly in hippocampal circuits. (17). In epilepsy, aberrant LTP, network hyperexcitability, and synaptic remodeling are central features (18). Similarly, in AD, synaptic dysfunction—marked by impaired LTP and LTD—is among the earliest manifestations leading to cognitive impairment (19, 20). This mechanistic overlap makes Alzheimer’s disease a relevant topic in reviews that aim to contextualize synaptic plasticity dysfunction within broader neurological processes. Moreover, epidemiological and experimental evidence have increasingly suggested a bidirectional relationship between epilepsy and Alzheimer’s disease. Chronic seizures have been associated with increased risk for AD, while mouse models of AD often exhibit spontaneous seizures or heightened seizure susceptibility (17, 21). These findings have prompted review authors to consider Alzheimer’s pathology in discussions about the mechanisms and clinical implications of epilepsy. Further reinforcing this connection, the emergence of “amyloid beta peptide” as a burst keyword in reviews not only confirms the relevance of Alzheimer’s disease to epilepsy research but also underscores a broader conceptual integration—where synaptic dysfunction, amyloid pathology, and seizure susceptibility are viewed as interrelated processes.

An important trend emerging from our bibliometric analysis is the increasing convergence of both review articles and original research on the molecular underpinnings of synaptic plasticity, particularly those involving neurotransmitters and their receptors. This is evidenced by the recent appearance of “glutamate” and “glutamate receptors” as burst keywords in review articles, and “NMDA” and “NMDA receptors” in original research articles. This pattern suggests a strategic shift in research emphasis. While earlier phases of epilepsy and synaptic plasticity research may have been dominated by descriptive neuroanatomy, electrophysiological observations, or systems-level models, the current focus is increasingly aligned with molecular and receptor-level mechanisms. Glutamate, as the principal excitatory neurotransmitter in the central nervous system, and NMDA receptors, as its critical mediators in synaptic plasticity and excitotoxicity, have emerged as central research targets (22).

In review literature, the burst of keywords like “glutamate” reflects a thematic consolidation, where scholars synthesize emerging data into neurochemical frameworks that connect synaptic plasticity with disease states such as epilepsy and neurodegeneration. These reviews often serve to highlight the relevance of neurotransmitter systems and predict future research directions. In original research, the shift toward specific receptor subtypes—particularly NMDA receptors—indicates that experimental investigations are now probing deeper into the molecular cascades involved in LTP, seizure initiation, and synaptic remodeling. The increasing frequency of terms like “NMDA” and “NMDA receptors” reflects not only technological advancements but also a growing emphasis on precision neuroscience, where identifying therapeutic targets at the receptor level is a priority. These findings point to a unified thematic evolution: both review and original research are progressively gravitating toward neurotransmitter signaling and receptor dynamics as core mechanisms underlying synaptic plasticity and epilepsy. This convergence may signal a maturation of the field, where interdisciplinary approaches—from cellular neurobiology to systems pharmacology—are increasingly integrated around common molecular denominators.

Hebbian and homeostatic plasticity are two fundamental mechanisms that play crucial roles in the regulation of neuronal excitability and synaptic strength. These processes are particularly relevant in the context of epilepsy. In epilepsy, the balance between excitatory and inhibitory signals in the brain is often disrupted, leading to excessive neuronal firing (23). Understanding how Hebbian and homeostatic plasticity contribute to this imbalance can provide insights into potential therapeutic strategies (24).

Hebbian plasticity, often summarized by the phrase “cells that fire together wire together,” involves the strengthening of synapses based on the correlation of pre- and postsynaptic activity. This form of plasticity can lead to increased excitability in neuronal networks, potentially contributing to the hyperexcitability observed in epileptic seizures. On the other hand, homeostatic plasticity acts as a stabilizing force, adjusting the intrinsic excitability of neurons to maintain overall network stability. This mechanism can counteract the effects of Hebbian plasticity by reducing synaptic strength or increasing inhibitory inputs when neuronal activity becomes too high (25, 26).

Research has shown that homeostatic plasticity can manifest through various mechanisms, such as the regulation of ion channel expression and function. For example, in the striatum, dopamine depletion leads to an increase in the intrinsic excitability of medium spiny neurons through the modulation of A-type potassium currents, representing a form of homeostatic plasticity that compensates for synaptic perturbations (23, 27). Similarly, in the cardiac ganglion of the crab, rapid compensatory interactions among potassium currents stabilize both intrinsic excitability and network function, highlighting the robustness of homeostatic mechanisms in maintaining neural network output (28).

In the context of epilepsy, these plasticity mechanisms may become dysregulated, leading to persistent changes in neuronal excitability and synaptic connectivity. Understanding the interplay between Hebbian and homeostatic plasticity in epilepsy could pave the way for novel therapeutic approaches aimed at restoring the balance of excitatory and inhibitory signals in the brain, potentially reducing seizure frequency and severity.

This study offers several unique advantages. Initially, it provides the inaugural systematic review of synaptic plasticity studies in epilepsy through bibliometric methods, delivering extensive insights for researchers focused on this area. Secondly, we utilized two bibliometric instruments concurrently for the study, such as VOSviewer and CiteSpace, which are well-known in the bibliometric domain, to guarantee an impartial data analysis procedure. In conclusion, bibliometric analysis provides deeper understanding of key areas and emerging trends than conventional reviews.

Naturally, this study has some limitations. First, it depends exclusively on information from the WoSCC database, which might miss pertinent research available in other databases. Second, we focused exclusively on studies published in English, thereby excluding potentially significant articles in other languages. Additionally, our study focused solely on two types of documents—reviews and articles—excluding other forms of publications such as books or conference papers from our bibliometric analysis.

## 5 Conclusion

This research offers insights into synaptic plasticity in epilepsy through visualization and bibliometric analysis. We examined the key areas of publication trends and emerging research topics. Regarding publication patterns, studies on synaptic plasticity related to epilepsy have shown a consistent rise. At present, the focus and emerging areas of review articles have shifted from long-term potentiation, astrocytes, and depression to gamma-aminobutyric acid, amyloid beta peptide, and glutamate receptors. Meanwhile, the focus and emerging areas of original research have shifted from synaptic reorganization, dentate granule cells, and messenger RNA to astrocytes, NMDA receptors, and long-term potentiation.

## Data Availability

The original contributions presented in the study are included in the article/Supplementary material, further inquiries can be directed to the corresponding author.

## Supplementary material

SUPPLEMENTARY FIGURE 1 **(A)** The dual-map overlay displays journals that publish research on synaptic plasticity in epilepsy (review publications). **(B)** The dual-map overlay displays journals that publish research on synaptic plasticity in epilepsy (original research publications). Journals that cite are shown on the left, while those being cited are on the right, with lines indicating citation connections.

SUPPLEMENTARY FIGURE 2 **(A)** Co-citation of cited references (review publications). **(B)** Co-citation of cited references (original research publications). **(C)** Co-citation of cited journals (review publications). **(D)** Co-citation of cited journals (original research publications).
