# Supplementary figures and images for: A bibliometric analysis of synaptic plasticity and epilepsy from 2003 to 2023
Source: Front Neurol. 2025 Jul 16;16:1533268. doi: 10.3389/fneur.2025.1533268 (PMC12307413; doi:10.3389/fneur.2025.1533268)

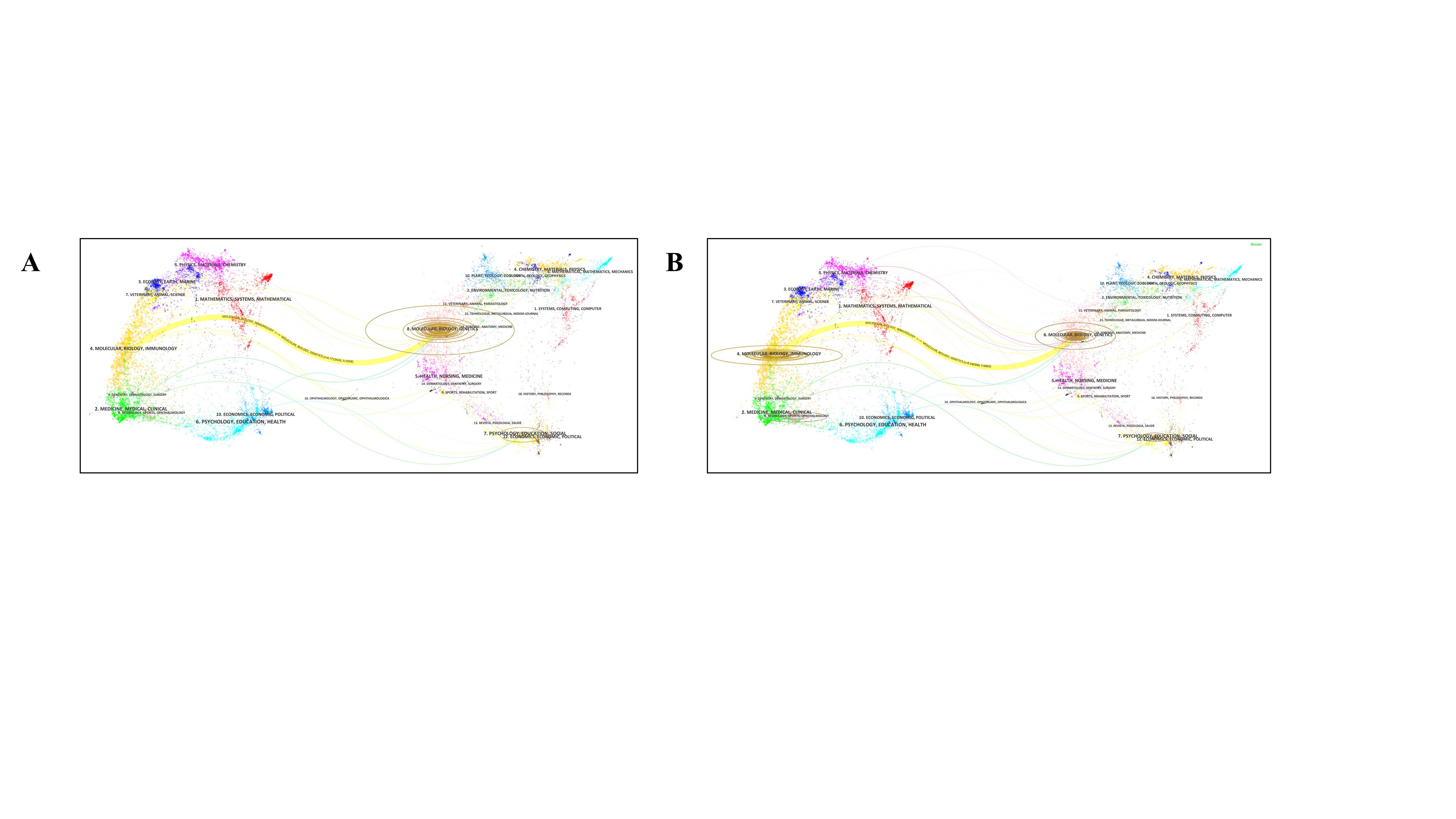

Supplement: SUPPLEMENTARY FIGURE 1 — (A) The dual-map overlay displays journals that publish research on synaptic plasticity in epilepsy (review publications). (B) The dual-map overlay displays journals that publish research on synaptic plasticity in epilepsy (original research publications). Journals that cite are shown on the left, while those being cited are on the right, with lines indicating citation connections. [file Image_1.tif]

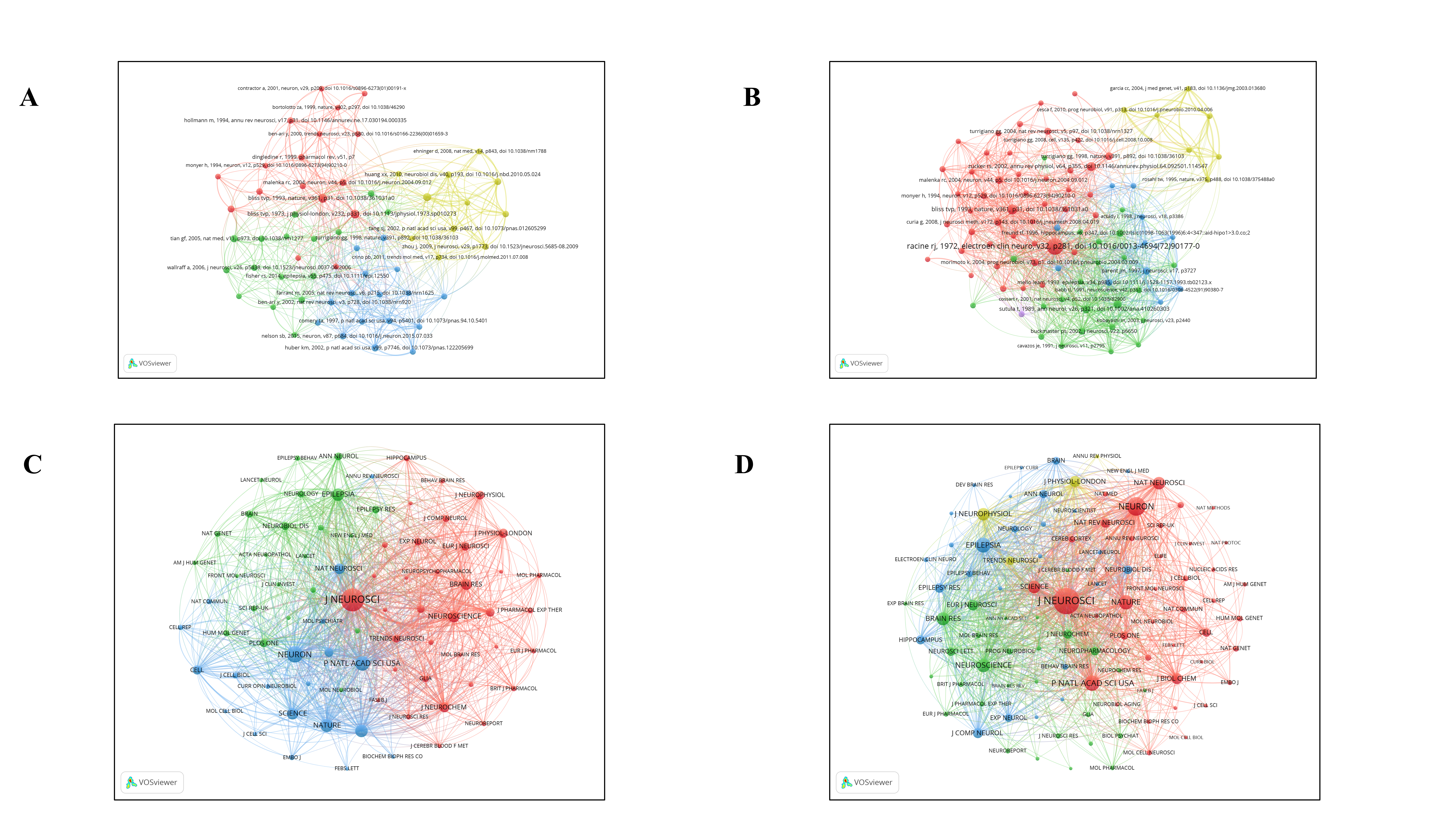

Supplement: SUPPLEMENTARY FIGURE 2 — (A) Co-citation of cited references (review publications). (B) Co-citation of cited references (original research publications). (C) Co-citation of cited journals (review publications). (D) Co-citation of cited journals (original research publications). [file Image_2.tif]
