# Supplementary material for: A bibliometric analysis of synaptic plasticity and epilepsy from 2003 to 2023
Source: Front Neurol. 2025 Jul 16;16:1533268. doi: 10.3389/fneur.2025.1533268 (PMC12307413; doi:10.3389/fneur.2025.1533268)
Supplement: Supplementary file 3 [file Supplementary_file_1.docx]

Table1. Top 10 co-cited references of review publications related to synaptic plasticity in epilepsy

| Rank | Co-cited reference | Co-cation |
| --- | --- | --- |
| 1 | bliss tvp, 1973, j physiol-london, v232, p331, doi 10.1113/jphysiol.1973.sp010273 | 19 |
| 2 | bliss tvp, 1993, nature, v361, p31, doi 10.1038/361031a0 | 19 |
| 3 | zeng lh, 2009, j neurosci, v29, p6964, doi 10.1523/jneurosci.0066-09.2009 | 16 |
| 4 | huber km, 2002, p natl acad sci usa, v99, p7746, doi 10.1073/pnas.122205699 | 13 |
| 5 | tian gf, 2005, nat med, v11, p973, doi 10.1038/nm1277 | 13 |
| 6 | malenka rc, 2004, neuron, v44, p5, doi 10.1016/j.neuron.2004.09.012 | 12 |
| 7 | meikle l, 2008, j neurosci, v28, p5422, doi 10.1523/jneurosci.0955-08.2008 | 12 |
| 8 | paoletti p, 2013, nat rev neurosci, v14, p383, doi 10.1038/nrn3504 | 12 |
| 9 | ben-ari y, 2002, nat rev neurosci, v3, p728, doi 10.1038/nrn920 | 11 |
| 10 | dingledine r, 1999, pharmacol rev, v51, p7 | 11 |

Table 2. Top 10 co-cited references of original research publications related to synaptic plasticity in epilepsy

| Rank | Co-cited reference | Co-cation |
| --- | --- | --- |
| 1 | racine rj, 1972, electroen clin neuro, v32, p281, doi 10.1016/0013-4694(72)90177-0 | 148 |
| 2 | bliss tvp, 1993, nature, v361, p31, doi 10.1038/361031a0 | 54 |
| 3 | zucker rs, 2002, annu rev physiol, v64, p355, doi 10.1146/annurev.physiol.64.092501.114547 | 43 |
| 4 | sutula t, 1989, ann neurol, v26, p321, doi 10.1002/ana.410260303 | 37 |
| 5 | tauck dl, 1985, j neurosci, v5, p1016 | 34 |
| 6 | curia g, 2008, j neurosci meth, v172, p143, doi 10.1016/j.jneumeth.2008.04.019 | 32 |
| 7 | morimoto k, 2004, prog neurobiol, v73, p1, doi 10.1016/j.pneurobio.2004.03.009 | 32 |
| 8 | malenka rc, 2004, neuron, v44, p5, doi 10.1016/j.neuron.2004.09.012 | 31 |
| 9 | turrigiano gg, 1998, nature, v391, p892, doi 10.1038/36103 | 31 |
| 10 | parent jm, 1997, j neurosci, v17, p3727 | 30 |

Table 3. Top 10 co-cited journals of review publications related to synaptic plasticity in epilepsy

| Rank | Source | Citations |
| --- | --- | --- |
| 1 | J NEUROSCI | 3518 |
| 2 | NEURON | 1744 |
| 3 | P NATL ACAD SCI USA | 1379 |
| 4 | NATURE | 1129 |
| 5 | EPILEPSIA | 1049 |
| 6 | J BIOL CHEM | 1037 |
| 7 | SCIENCE | 906 |
| 8 | NEUROSCIENCE | 865 |
| 9 | BRAIN RES | 813 |
| 10 | NAT NEUROSCI | 748 |

Table 4. Top 10 co-cited journals of original research publications related to synaptic plasticity in epilepsy

| Rank | Source | Citations |
| --- | --- | --- |
| 1 | J NEUROSCI | 5898 |
| 2 | NEURON | 3014 |
| 3 | P NATL ACAD SCI USA | 2016 |
| 4 | EPILEPSIA | 1935 |
| 5 | NATURE | 1756 |
| 6 | NEUROSCIENCE | 1594 |
| 7 | BRAIN RES | 1421 |
| 8 | J NEUROPHYSIOL | 1370 |
| 9 | SCIENCE | 1332 |
| 10 | NAT NEUROSCI | 1288 |
